# International Stem Cell Collaboration: How Disparate Policies between the United States and the United Kingdom Impact Research

**DOI:** 10.1371/journal.pone.0017684

**Published:** 2011-03-08

**Authors:** Jingyuan Luo, Jesse M. Flynn, Rachel E. Solnick, Elaine Howard Ecklund, Kirstin R. W. Matthews

**Affiliations:** 1 Science and Technology Policy Program, James A. Baker III Institute for Public Policy, Rice University, Houston, Texas, United States of America; 2 Department of Biochemistry and Molecular Biology, The University of Texas M. D. Anderson Cancer Center, Houston, Texas, United States of America; 3 Department of Sociology, Rice University, Houston, Texas, United States of America; University of Southern California, United States of America

## Abstract

As the scientific community globalizes, it is increasingly important to understand the effects of international collaboration on the quality and quantity of research produced. While it is generally assumed that international collaboration enhances the quality of research, this phenomenon is not well examined. Stem cell research is unique in that it is both politically charged and a research area that often generates international collaborations, making it an ideal case through which to examine international collaborations. Furthermore, with promising medical applications, the research area is dynamic and responsive to a globalizing science environment. Thus, studying international collaborations in stem cell research elucidates the role of existing international networks in promoting quality research, as well as the effects that disparate national policies might have on research. This study examined the impact of collaboration on publication significance in the United States and the United Kingdom, world leaders in stem cell research with disparate policies. We reviewed publications by US and UK authors from 2008, along with their citation rates and the political factors that may have contributed to the number of international collaborations. The data demonstrated that international collaborations significantly increased an article's impact for UK and US investigators. While this applied to UK authors whether they were corresponding or secondary, this effect was most significant for US authors who were corresponding authors. While the UK exhibited a higher proportion of international publications than the US, this difference was consistent with overall trends in international scientific collaboration. The findings suggested that national stem cell policy differences and regulatory mechanisms driving international stem cell research in the US and UK did not affect the frequency of international collaborations, or even the countries with which the US and UK most often collaborated. Geographical and traditional collaborative relationships were the predominate considerations in establishing international collaborations.

## Introduction

The scientific community is increasingly global; new scientific results are disseminated worldwide within hours of publication. From 1998 to 2008, the absolute number of internationally co-authored publications in science (including social sciences) and engineering almost doubled from 98,424 to 180,783 (representing over 20% of total publications in 2008) [1]. As the number of internationally co-authored journal articles proliferates, it is imperative to understand the impact that cross-border collaborations may have on the quality, productivity, and effectiveness of the research produced.

Previous research has established that articles with authors from multiple countries were cited twice as frequently as publications authored by scientists working at a single institution or within a single country [2], [3]. Moreover, scholarship reveals that multi-institutional collaboration, particularly collaborations that involve institutions in different nations, also increased citation rate [2], [4], [5]. Reviewing overall science and engineering publications in 2008, 43% of internationally co-authored papers included US-based researchers. Germany and the UK shared the next highest percentage, with 19% each [1]. While Germany shares the second highest percentage of stem cell publications, Germany's stem cell policy is similar to US policy from 2001 to 2008, with restrictions on human embryonic stem cell (hESC) research based on the time the cells were derived [6]. In contrast to the US, the UK has a more permissive approach within a highly detailed regulatory system. The UK and US were therefore selected for this study due to their policy differences and high frequency of collaborations. Examining the impact of collaboration in these two countries will highlight the effects of disparate policy regimes on scientific research.

Due to policy disparities between nations and extant international research networks, stem cell research is the ideal research area for a study of the impact of international collaboration. Stem cell research—embryonic, cord blood, adult, and induced pluripotent (iPS)—has the potential to revolutionize medicine and provide scientists with an improved understanding of cell development and specialization. Prior studies of stem cell research in the Middle East suggest that international collaborations resulted in stem cell publications with a higher citation rate than articles published by a single nation from the region [7]. However, no studies of collaborations in stem cell research have been conducted on publications with co-authors from two of the leading countries in this field, the US and the UK. Potential stem cell therapies offer the promise of possible treatments for debilitating injuries, e.g., spinal cord and brain trauma, and cures for debilitating diseases and conditions such as Parkinson's disease, diabetes, and blindness [8], [9]. At the same time, progress on hESC research in the US, in particular, is limited by policy restrictions and political uncertainties. For these reasons, stem cell research presents a unique opportunity not only to study international collaboration per se, but collaboration under very different policy regimes.

Research from the US and the UK involving hESCs is conducted under quite different legislative policies. US policy on stem cell research has developed sporadically, has historically been more inhibitive than supportive, and has included only federal government funding of the research. By contrast, UK policy has developed in a more permissive, although highly regulated, manner [10]–[12].

Since 1978, following the first successful *in vitro* fertilization (IVF) birth, the UK has progressively built upon its policy. The first set of recommendations governing embryo research appeared in the Warnock Report, which was released in 1984 [13]. This report encouraged regulated embryo research. It also limited embryo research to the first 14 days of development, a standard now applied worldwide. In 1990, legislation to regulate embryo research, the Human Fertilization and Embryology (HFE) Act, was passed in the UK. This established the Human Fertilization and Embryology Authority (HFEA), which monitors and grants licenses for all embryo research, regardless of the source of funding [14]. As science progressed, the UK revised the HFE Act in 2001 and 2008 to take into account scientific advances such as the creation of the first hESC line [15]. In 2003, in order to provide storage for hESC lines created using HFEA licenses, the UK also launched the UK Stem Cell Bank. The UK has identified new funds specifically for stem cell research, apparently with little controversy [16]. It has also encouraged international collaborations through its commonwealth offices.

In contrast to the UK, the US has been slow to adopt a comprehensive human embryo policy. In 1978, President Carter established the Ethics Advisory Board to monitor embryonic research, but after the election of President Reagan, the board never met and never approved a single project [10]. The board was eventually dismantled by the Clinton administration, which planned to provide funding for human embryo research. However, while the Clinton policy was being put in place, Congress passed the Dickey-Wicker amendment. This amendment, which has been added to the funding bill for the National Institutes of Health (NIH) each year since 1995, forbids the use of federal funds for research in which a human embryo is destroyed or subjected to risk of injury or death [17]. The amendment also bans federal funding for the creation of hESC cell lines, which were first derived in 1998, four years after the amendment was enacted. NIH carefully studied the Dickey-Wicker amendment and determined that it would permit funding of hESC research on stem cell lines derived from donated IVF embryos supported by non-federal funds. However, before any such research could be funded, Clinton left office. Limited hESC research funded by the federal government was allowed under President G.W. Bush, but only for lines created before August 9, 2001 (21 hESC lines) [18]. In 2009, under the Obama administration, federal funding for stem cell lines created after August 9, 2001, became available to researchers, but under strict guidelines. It is still not possible to use federal funds for the derivation of hESC lines regardless of the method used. In the US, there is no restriction on hESC research or even reproductive cloning as long as no federal funds are involved. Any potential stem cell therapy, however, requires demonstration of safety and efficacy and final approval by the US Food and Drug Administration (FDA).

The lack of a coherent approach and set of policies for human embryo and hESC research in the US has resulted in the most recent policy crisis. On August 23, 2010, US District Judge Royce Lamberth ruled that the Dickey-Wicker amendment prohibited federal funding of any hESC for research [19]. This decision translated into an immediate injunction halting research at the NIH (both intramural and extramural research) and impacted grant decisions worth $140 million [20]. Approximately two weeks later, on September 9, 2010, a federal appeals court lifted the injunction and agreed to listen to arguments for and against the ban. The court still has not ruled on the case itself. With policy implications ranging from a permanent injunction or ban of hESC research to possible congressional legislation explicitly permitting federal funding of hESC, the current precarious nature of hESC research in the United States highlights the difficulties scientists face and serves as a possible barrier to international collaborations involving US researchers.

In addition to policy disparities among nations, a second aspect of stem cell research that makes it an ideal research area for the study of international collaborations is its intrinsically international nature. This is evident in the various existing organizations that facilitate the exchange of research findings and policy information, e.g., the International Society for Stem Cell Research (www.isscr.org), the International Stem Cell Forum (www.stem-cell-forum.net), and the International Consortium of Stem Cell Networks (www.stemcellconsortium.org). These international organizations formed while the field was still young. This presents the opportunity to study a field that is characterized by high levels of international collaborative research from the start.

The goal of this article is to identify the impact of international collaboration on citation rates in stem cell research in the US and the UK. We specifically examined if international collaborations increase citation rates, and if differing research regulations and legislations affect the frequency of collaboration. Publications were examined from the US and the UK for the year 2008. Citation rates, country attribution, and collaborations were recorded for each article. In addition, we analyzed the political landscape and other driving factors, which may have contributed to the number and impact of these international collaborations. The data confirmed that, for both the US and the UK, international collaborations significantly increased an article's impact (as measured by citation rates). It also demonstrated that the UK participated in a higher percentage of international publications than the US, which is congruent with trends of international collaborations in all sciences [1]. Four of the top five collaborators with the US were Germany, UK, Canada, and China, countries that were consistent across all fields of science, indicating that traditional collaborative relationships also predominated in international stem cell collaborations [1]. This suggests that policies and regulatory differences in the UK and US did not influence the frequency of international collaborations nor the countries that the US and UK collaborate with in stem cell research.

## Methods

### Article Collection and Sorting

Publications used for this study were extracted from Thomas Reuters' Institute of Scientific Information (ISI) Science Citation Index. Stem cell research articles were identified by entering the search string: TS = (“stem cell*”) AND CU = Respective Country AND Document Type = (Article), and setting the time period to 2008. The UK data was collected by searching for articles from England, Scotland, Wales, and Northern Ireland. All articles for both the US and UK were collected on December 8, 2010. Records were sorted to eliminate articles (UK or US) when the corresponding author's address listed did not match the address of that individual when the research was conducted. Also excluded in this study were publications not indexed by ISI, non-English language journals, and articles that did not use stem cells in an experimental context—such as reviews, book chapters, abstracts, and conference proceedings. Self-citations were not removed.

### Categorical Assignment of Articles

The articles collected were divided into three categories: independent (Indep), international-corresponding (Intl-C), and international-secondary (Intl-S). Articles were considered independent papers only when researchers from either the US or the UK were listed. Articles were considered international-corresponding works when a scientist from the US or the UK was the corresponding author, and the article listed authors from two or more countries. The corresponding author was determined from the reprint address. Articles that listed authors from two or more countries—one of which was the US or the UK—but did not list a scientist from one of these countries as the corresponding author were considered international-secondary papers.

### Statistical Analysis

Unpaired, two-tailed, Student's t-tests assuming equal variance were conducted with the alpha value set to 0.05.

## Results

**Figure 1:**
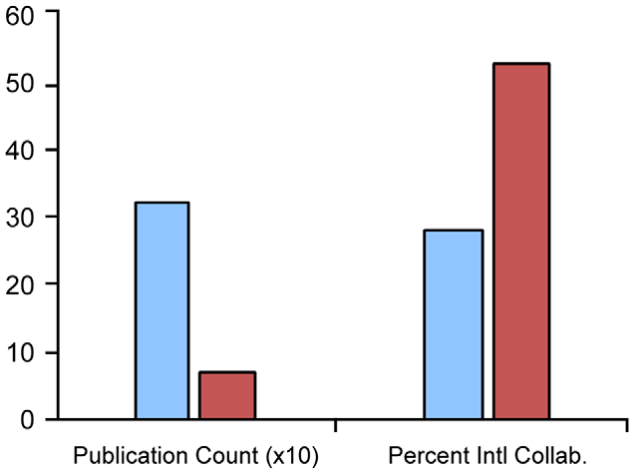
Comparison of US and UK articles. While the United Kingdom (red) collaborates proportionally more than the United States (blue), with 53.4% of their publications the result of international collaborations versus 27.1% in the United States, the United States produced a higher absolute number of publications (3176 versus 616 in the United Kingdom).

A literature search of 2008 publications generated 3176 articles that listed at least one US scientist as an author, and a total of 616 papers that listed at least one UK scientist as an author (Figure 1). While US researchers published over five times more often than UK researchers in absolute numbers, the publication rates per million inhabitants were very similar—10.2 articles per million individuals for the US and 10.0 articles per million individuals for the UK [21], [22].

**Figure 2:**
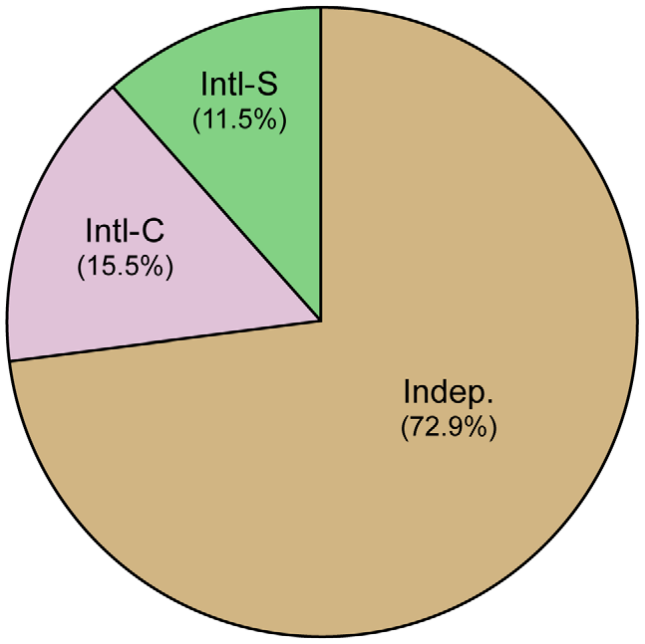
Collaborative status of US articles. Of the 3176 papers generated by the US, 15.6% (494) were international-corresponding (Intl-C), 11.5% (366) were international-secondary (Intl-S), and 72.9% (2316) were independent papers (Indep).

**Figure 3:**
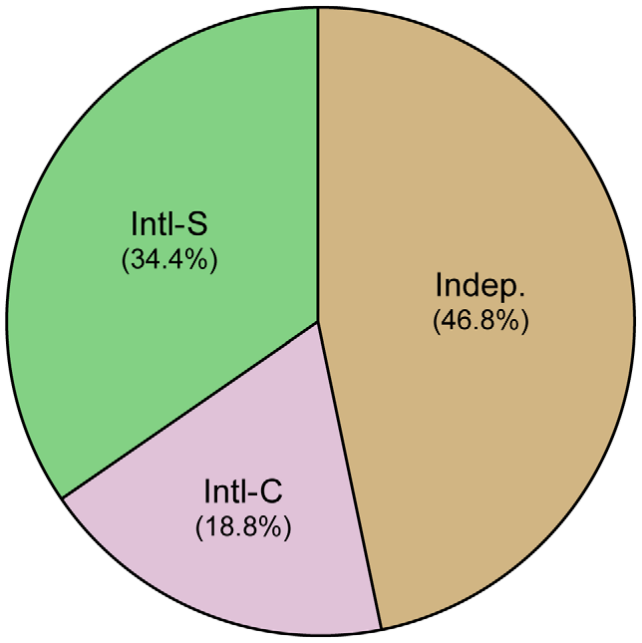
Collaborative status of UK articles. Of the 616 articles pulled for the UK, 18.8% (116) were international-corresponding (Intl-C), 34.4% (212) were international-secondary (Intl-S), 46.8% (288) were independent (Indep).

The collaboration rates for these publications were subsequently investigated. Proportionally, the UK engaged in appreciably more collaborative research in both a corresponding and secondary capacity—18.8% and 34.4% (total 53.2%), respectively, compared with 15.5 and 11.5% (total 27.1%) for the US (Figures 2 and 3). The data is consistent with overall trends in collaborations which show the US collaboration rates in all sciences (including engineering and social science research) at 30% and the UK at 49% [1].

The citation rates for these articles broadly ranged from 0 to 196 citations for UK publications and from 0 to 592 citations for US articles (Table 1). With the exception of a few articles, the majority of publications from both nations were cited fewer than 10 times. Sixty-one percent of the publications involving the US or UK received 10 or fewer citations, with 12% of them receiving 0–1 citations. The distribution of papers based on the citation rate were similar for the US and UK for rates under 100 citations. Papers with 100+ citations made up 1.8% (57 papers) of total US publications while they were only 0.81% (5 papers) of total UK publications. No UK papers had over 200 citations, but 19 US papers did.

**Table 1:** Distribution of articles by citation rate and percentage of total for US and UK.

# of Citations	US	% of Total US	UK	% of Total UK
**0**	176	5.54	34	5.52
**1–5**	1007	31.7	200	32.5
**6–10**	745	23.5	140	22.7
**11–50**	1082	34.1	220	35.7
**51–100**	109	3.43	17	2.76
**101–200**	38	1.20	5	0.81
**201–500**	18	0.57	0	0
**501+**	1	0.03	0	0

**Figure 4:**
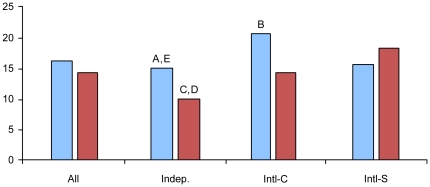
Average number of citations for US and UK paper. US publications (blue) UK publications (red) were evaluated in four categories: overall citation rate (All); independent (Indep); international-corresponding (Intl-C); and international-secondary (Intl-S). Significant differences statistically were seen between: (A) US Indep vs. Intl-C, p<0.01; (b) US Intl-C vs Intl-S, p = 0.04; (C) UK Indep vs.Intl-C, p = 0.01; (D) UK Indep vs. Intl-S, p<0.01; and (E) US Indep vs. UK Indep, p<0.01.

Overall citations from the US papers were slightly higher than UK papers, (15.9 versus 13.6), but the results were not statistically significant. Reviewing data for each country, citation rates for UK articles were significantly higher when the paper was the result of an international collaboration rather than independently produced by the UK. UK-independent articles averaged 10.1 citations while articles listing a UK scientist as the international-corresponding or an international-secondary author averaged 13.8 (p = 0.01) and 18.4 citations (p<0.01), respectively (Figure 4). The increased citation rate of articles by US scientists collaborating with international co-authors was slightly less dramatic, but still statistically significant (p<0.01 for papers on which the US was the corresponding author versus US independent papers). US-independent articles averaged 15.0 citations, and publications listing a US scientist as the international-corresponding and international-secondary author averaged 20.3 and 15.3 citations, respectively. While the citation rate was slightly increased for international papers on which a US scientist was a secondary author, this difference was not found to be statistically significant, indicating that it is not as beneficial for US authors to be secondary contributors. These figures suggest that scientists in both the UK and US produce higher-impact stem cell research when collaborating with foreign counterparts. But US scientists find a more dramatic increase in citation rates when they are corresponding authors and the UK scientists had the highest rate for articles as secondary authors.

The publications on which US and UK researchers had international co-authors were then analyzed to determine the countries with which the US and UK scientists most often collaborated (Table 2 and 3). The United States had 52 collaborating countries with the top ten collaborators representing 60.0% of the international publications. The citation average of the top ten collaborators ranged from 9.99 (China) to 26.4 (Spain). In contrast, the UK had 42 collaborating countries. The top 10 represented 52.4% of the international publications, and the citation average of the top ten collaborators ranged from 15.0 (Switzerland) to 40.3 (Canada). All of the top collaborators (except for China and South Korea) resulted in average citations which were higher than the citations of papers with US- or UK-only authorships.

**Table 2:** Top 10 countries with collaborated with the United States.

Country	Average Citations	Total # of Publications	% Total
Germany	22.89	138	16.0
Japan	23.17	113	13.1
UK	21.07	105	12.2
Canada	18.40	101	11.7
China	9.99	100	11.6
Italy	17.76	71	8.26
France	17.50	48	5.58
South Korea	10.77	47	5.47
Netherlands	22.32	41	4.77
Spain	26.41	39	4.53
Remaining 42 Countries	11.90	344	40.0

**Table 3:** Top 10 countries with collaborated with the United Kingdom.

Country	Average Citations	Total # of Publications	% Total
USA	21.23	128	39.0
Germany	22.24	70	21.3
Italy	18.62	39	11.9
Netherlands	17.50	40	12.2
France	23.45	33	10.1
Switzerland	14.96	28	8.54
Sweden	21.72	25	7.62
Spain	27.35	23	7.01
Canada	40.32	22	6.71
Japan	22.30	20	6.10
Remaining 32 Countries	17.56	156	47.6

## Discussion

As scientific research becomes increasingly global, it is important to understand how increased international collaboration affects the impact of the research produced. Stem cell research is an ideal area for the study of scientific globalization. It is heavily influenced by policy decisions, which differ markedly across borders, and is the subject of many international dialogues and networks. US and UK articles were chosen for this study because these two countries have disparate policy environments, yet are both leaders in stem cell research.

Here, we used citation rates as a measure of impact to evaluate the significance of publications. Citation rates are one quantitative measure of publication quality that is used across disciplines and across national contexts. We recognize this is not the only way, or even the best way, to measure quality, but that it is the simplest, taking into account the scale of research in the US and UK. This study demonstrated that UK stem cell researchers engaged in international collaborations proportionally more frequently than US investigators. We have also shown that stem cell articles resulting from UK and US international collaborations were cited significantly more often than those generated by solely UK or US investigators. This increase was not as large for the US as it was for the UK, yet both countries benefited from international collaborations.

There are many possible reasons why collaborative publications garner higher average rates of citation than single author, institution, or country publications. Collaboration can be beneficial through the sharing of resources, ideas, expertise, and institutions. Collaborations, especially international collaborations, might also provide a researcher, and consequently his or her work, more exposure within the field. Collaborating with a well-established laboratory may provide a mechanism for newer and less well-known researchers to network within the stem cell research community. It has been proposed that the number of authors on a paper increases the citation rate simply due to increased self-citation; however, studies have indicated that this does not apply to all fields and, thus far, the phenomenon has not been examined in international collaborations [4], [23]–[26].

By examining citation rates in international collaborations, the present study is a first step in understanding the possible benefits of international collaboration. Some fields are more likely to encourage and engage in international collaboration. Similarly, some researchers are more likely than others to pursue international collaborations; these individuals may be well established in their respective field and have both the funding and institutional support to pursue collaborative initiatives. With so many variables, it is difficult to identify with any certainty the aspects of collaborations, particularly international collaborations, which result in higher citation rates. The goal of the paper, however, was not to provide an explanation of how collaborations increase citation rates. Instead, we sought to determine if this trend has persisted in biomedical research, specifically stem cell research, in two nations with mature science and technology research and development programs, and how policy differences affected the nature of stem cell collaboration.

The data demonstrated that it was beneficial for UK researchers to participate in these collaborations, as the number of citations increased significantly when a UK researcher was the corresponding author or a secondary author. While it was beneficial for US-based researchers to engage in international collaborations, it appeared that the benefit was most salient when the US researcher took the role of corresponding author. The higher citation rates of papers by US researchers in the position of corresponding authors may be the result of the large biomedical research infrastructure and numerous funding opportunities that exist in the US.

There are many possible factors that account for the increased participation by UK investigators in international collaborations relative to the US. The UK has a multitude of countries in its immediate proximity with which to collaborate, many of which boast well-developed biomedical research facilities. Although the US is geographically larger than the UK, the pool of neighboring countries with which its investigators can easily collaborate is far more limited.

Additionally, the UK has fewer scientists per capita than the US; 0.04% of UK inhabitants hold scientific degrees as opposed to 0.07% of the US population (2,301,000 UK scientists versus 22,630,000 US scientists) [21], [22], [27], [28]. This population difference could lead to a lower rate of domestic co-authorships in the UK, as UK researchers do not have as many options as US researchers when selecting a domestic partner for collaboration [3]. While previous research concluded that international collaborations could more often be traced to historical and linguistic factors than the number of scientists in a country, the population difference can nevertheless serve as one factor encouraging increased international collaborations [2].

Another reason UK scientists collaborate internationally might be that funding levels for stem cell research are lower in the UK. In 2007, the NIH invested $968 million in stem cell research in the US, approximately $3.12 per individual (total US population) [20], [26]. In contrast, the two UK research councils that fund stem cell research, the Medical Research Council (MRC) and the Biotechnology and Biological Sciences Research Council (BBSRC), spent £33.9million ($69.5 million), or approximately $1.21 per UK resident [22], [29]. The United Kingdom's overall funding for stem cell research in 2007, including federal and private funding, was £62 million ($122.5 million), or approximately $2.05 per individual [22], [29]. This was substantially less than the US's NIH funding, and it is likely that support from the European Union (EU) compensates for part of the difference.

The EU, in which the UK is a member, heavily promotes collaborative research and could be another contributing factor to the proportionally higher rate of UK investigators' international collaboration. In 2000, the EU took steps to unify research efforts with the creation of the European Research Area [30]. While stem cell research, particularly hESC research, is a controversial topic in Europe, the EU has funded and continues to fund stem cell research. Under the Sixth Framework Program (2002–2006), the EU specifically targeted collaborative research projects in stem cell research and despite considerable controversy, has committed to include stem cell research as a part of its €54 billion (approximately $69 billion) research budget for the Seventh Program Framework (FP7), in effect from 2007 to 2013 [31]. As collaboration and networking in biomedical research are increasingly important, the bulk of the FP7 will go toward collaborative research in the EU and beyond through a variety of funding schemes, including collaborative projects (between member states), coordination/support actions, and joint technology initiatives [32], [33].

The US and UK shared seven of the same top 10 collaborators (although in a different order): Germany, Italy, Netherlands, France, Spain, Canada, and Japan (see Tables 2 and 3). While the US was the top collaborator for the UK, the UK ranked third on the list for the US. The countries that did not overlap were China and South Korea (top collaborators with the US) and Switzerland and Sweden (top collaborators with the UK). The US results were consistent with previous studies of co-authorship by the National Science Foundation, which included all publications from natural science, social science, and engineering research in 2008. That report determined that US authors were most likely to collaborate with the UK (13.9%), Germany (12.7%), Canada (12.0%), and China (10.4%) [1].

Japan and Germany were two major collaborators with both the US and UK. While progress on hESC in Japan has proceeded slowly due to the need to establish a regulatory framework, Japan's stem cell research policies are largely permissive. As a result, large investments have been made in centers such as the RIKEN Center for Developmental Biology (www.cdb.riken.jo.jp) and the Institute for Frontier Medical Sciences at Kyoto University (www.frontier.kyoto-u.ac.jp) [34]. With a 2004 legislation change that permitted the creation of human embryos for stem cell research and the development of induced pluripotent stem (iPS) cells by Shinya Yamanaka's team at the Kyoto University Institute for Frontier Medical Sciences, the climate of stem cell research in Japan has become very advanced, thus attracting many international collaborators [35].

Germany, unlike Japan, has a fairly restrictive stem cell research policy, and bans the production of hESC lines. The loophole to this policy is that hESCs may be imported; this was directly addressed in Germany's 2002 Stem Cell Act, which permitted the use of stem cells created before January 1, 2002 for high-ranking research objectives [36]. Despite these restrictions, the German Federal Ministry of Education and Research (BMBF) as well as the German Science Foundation (DFG) have invested heavily, approximately €230 million ($300 million) since 1990 in regenerative medicine [36]. Germany is also currently one of the only six countries collaborating with the California Institute for Regenerative Medicine (CIRM), with the UK, Canada, Australia, Spain, and Japan being the others [37]. In addition to these efforts, various German grant organizations such as the Alexander von Humboldt Foundation and the DFG have targeted international research cooperation, thus helping to account for Germany's high rate of collaboration with the UK and the US [38]. This is also consistent with Germany being the top international collaborator with the US for 2008 in all fields of science and engineering research [1].

Of the top nations collaborating with the US or UK, the only non-overlapping countries were China and South Korea (both US collaborations) and Switzerland and Sweden (both UK collaborators). Asian collaborations are common for US researchers for all sciences according to the National Science and Engineering Indicators [1]. China does not object to the use of hESCs and permits the production of new hESC lines as well as therapeutic cloning [39]. South Korea also places a high priority on stem cell research and allows therapeutic cloning [40]. Furthermore, many Asian students study abroad in the US, which could lead to collaborations if they return home [41]. But unlike the other countries in the top ten for the US, Chinese and South Korean collaborations resulted in average citations (9.99 for China and 10.77 for South Korea) which were lower than the average of US-only publications (15.01).

Investigators in the UK, on the other hand, likely collaborated more often with Switzerland and Sweden because of their proximity, their membership in the Council of Europe, and their permissive approach toward stem cell research. Both Switzerland and Sweden allow for the derivation of human embryonic stem cell lines, and Sweden also allows therapeutic cloning [8], [42]. In 2004, a national referendum was put forth in Switzerland, with two-thirds of the voters deciding to support embryonic stem cell research [43]. Sweden also has a well-established biomedical industry with public and political support for stem cell research. Lines created from discarded embryos as well as through somatic cell nuclear transfer are permitted [44]. Two of the human embryonic stem cell lines approved by the National Institutes of Health for use in US federal government funded research are from the company Cellartis AB in Sweden [45].

This study is an initial investigation of the impact of international collaboration on publications in stem cell research. The results indicated that international collaborations, on average, significantly increased article citation rates (our metric for impact) for the UK and US investigators. Additional research is necessary to address the mechanisms of a successful collaboration by examining the extant research networks and elucidating best practice methodology to improve these interactions. Although citation rate was taken as an appropriate measure of publication quality for this study, additional indicators of quality and significance—such as scientific honors and awards, research funding, patents and infrastructure—should also be considered in determining the impact of international collaboration. As research globalizes and national funding agencies reward collaborative efforts, understanding the characteristics of a successful collaboration is crucial to maximizing the resources available for stem cell research and advancing this scientific field.
